# Nearest transfer effects of working memory training: A comparison of two programs focused on working memory updating

**DOI:** 10.1371/journal.pone.0211321

**Published:** 2019-02-13

**Authors:** Rocío Linares, Erika Borella, Mª Teresa Lechuga, Barbara Carretti, Santiago Pelegrina

**Affiliations:** 1 Department of Psychology, University of Jaen, Jaén, Spain; 2 Department of General Psychology, University of Padova, Padova, Italy; Liverpool John Moores University, UNITED KINGDOM

## Abstract

This study analyzed the mechanisms involved in possible transfer effects for two different working memory updating (WMU) training programs administered to young adults and based on two updating paradigms: *n*-back and arithmetical updating. The influence of practice distribution on transfer effects was also explored by including two training regimens: massed and spaced practice. Performance on different WMU tasks more or less structurally similar to the tasks used in the training was assessed to analyze the nearest transfer effects. Near and far transfer effects were tested using complex working memory (WM) and fluid intelligence tasks. The results showed that the WMU training produced gains in only some of the WMU tasks structurally similar to those used in the training, not in those lacking the same structure, or in WM or fluid intelligence tasks. These limited nearest transfer effects suggest that gains could be due to the acquisition of a specific strategy appropriate for the task during the training rather than to any improvement in the updating process per se. Performance did not differ depending on the training regimen.

## Introduction

Working memory updating (WMU) is a fundamental cognitive function that enables us to modify stored information to adapt to new environmental demands. Its role in predicting fluid intelligence [1–3], and its relationship to academic achievement [4–6] have prompted numerous studies on the extent to which WMU training effects may generalize to other tasks dissimilar to those used in the training.

Gains induced by training in other measures not directly trained are known as transfer effects. There are various types of transfer effect depending on the level of generalization [7]. In the context of WMU training procedures, *nearest* transfer indicates improvements in other versions of the task used in the training and in other WMU tasks; *near* transfer refers to enhancements in other abilities closely associated with WMU, such as working memory (WM); and *far* transfer refers to gains in other abilities less, but still, related to WMU, such as fluid intelligence, attention, and switching [8].

Different paradigms have been used in WMU training studies to investigate these transfer effects. Perhaps the most frequently applied WMU paradigm has been the *n*-back, a task requiring that participants indicate whether the current stimuli match those presented *n* trials previously. Mixed results have been reported regarding transfer effects of this training task. Some training studies found training gains in other versions of the task used in the training that involved different stimulus material [9, 10]. The performance benefits spread to other WMU tasks [11], to classical complex WM tasks (e.g., [12]), and even to tasks that tap other less-related abilities. For example, gains were found in verbal fluency [13], fluid intelligence [8], and task switching [14]. On the other hand, a number of studies failed to show near transfer to other WM tasks [8, 9, 11], or far transfer to other abilities, such as fluid intelligence [14, 15] or inhibitory control [15, 16]. Some studies even failed to find transfer effects of any kind [17]. Some recent meta-analyses conducted to address these previous inconsistent results indicated that *n*-back training could induce far transfer effects, on fluid intelligence, for example ([18, 19], for older adults). Others were less conclusive, however [20–24]. The effectiveness of training based on the *n*-back task in eliciting transfer to other different tasks thus remains unclear.

A number of studies, that have used paradigms other than the *n*-back to train WMU, has found that nearest transfer effects are more common than near or far transfer effects. For example, using the running memory paradigm as a training task, different authors demonstrated nearest transfer effects on other WMU tasks, such as the *n*-back task [25–28], and far transfer to episodic memory tasks [27]. They found no near transfer to WM span tasks [27], however, nor any far transfer to selective attention [26], perceptual speed, fluid intelligence, verbal fluency, or other episodic memory measures [27]. In a recent study, Linares et al. [29] trained participants on numerical WMU tasks, and found nearest transfer to other WMU tasks structurally similar to those used in the training. The authors saw no far transfer to fluid intelligence or near transfer to complex WM tasks, however, nor even any nearest transfer to other WMU tasks dissimilar to those used in the training.

### Transfer mechanisms in training studies

These inconsistent results regarding the effectiveness of WMU training make it necessary to understand not only when and how transfer effects are observed, but also why they are obtained [23, 30]. Transfer effects may be due to a variety of mechanisms [30, 31], which have rarely been examined, and they warrant closer investigation to better capture the nature of training-related gains, and why they may or may not occur.

Morrison and Chein [31], for instance, distinguished between two different approaches to WM training: core training and strategy training. In the former, demanding WM tasks are administered to improve WM domain-general mechanisms, and the procedure used makes the development and use of strategies almost impossible. In the latter, participants are given extensive practice with tasks to which a certain strategy may be applied. Transfer effects may be driven by a different mechanism in each of the two training approaches: the strengthening of a process shared by different tasks; or the learning of a strategy suitable for applying to different tasks. Similar mechanisms to explain the transfer effects of WM training were also considered by von Bastian and Oberauer [30]. Training may improve WM capacity, for instance, or, instead increase WM efficiency through a more effective use of strategies. These possibilities are described in more detail below.

A first possibility is that the training can bring improvements thanks to a strengthening of the process used in the training. In this case, transfer effects would be likely to depend on the processes shared by trained and transfer tasks [32, 33]. Thus, a broad transfer across different tasks tapping the same process could be expected, whatever the structure of the tasks (i.e., the demands of the tasks, as detailed below). The greater the overlap between the cognitive abilities engaged in the trained process and the transfer tasks, the greater the likelihood of obtaining transfer effects. In support of this idea, Jaeggi et al. [32] found that *n*-back training produced far transfer effects on fluid intelligence measures, which share a good deal of variance with WMU, but no near transfer to measures of WM capacity, which share little variance with WMU (see also [34]). This is consistent with the idea that transfer occurs mainly when training and transfer tasks activate overlapping brain regions [26, 35–37].

A second possibility is that training may induce the acquisition of specific skills or strategies for trained tasks. If this is true, then we can expect to see transfer limited to structurally similar tasks [30] that share the same main subprocesses and can be completed following similar steps, despite some task-specific features. In other words, tasks are structurally similar when: information is presented in the same way (e.g., displayed in boxes); the response mode is similar (e.g., a response is required after each stimulus has been presented); and the tasks’ underlying cognitive processes require the same procedural steps (e.g., focusing first on the item to be updated, then retrieving the previous value related to the context, then modifying the item, and so on) [29]. Such structurally similar tasks would immediately facilitate the use of a strategy learned during the training. In fact, some authors have attributed near transfer to the acquisition of a strategy ([9, 29, 38]; see [21, 39] for a review) For example, Laine et al. [40] only found transfer to structurally similar untrained tasks (to which the same strategy could be applied), not to structurally different tasks. Some far transfer effects might also be feasibly explained by the acquisition of a general strategic approach applicable to other processes. For example, strategically focusing on a subset of items is an approach that can be applied to tasks that involve different processes [30].

To date, few studies have explicitly examined the mechanisms behind transfer effects. The present study aimed to examine the conditions that might prompt transfer effects: the strengthening of a common underlying cognitive process; or the acquisition of strategies suitable for approaching similar tasks. To our knowledge, ours is the first training study to simultaneously investigate both these possible explanations for transfer effects by implementing and comparing two different training programs based on two updating paradigms.

### Potential influences of practice distribution on training

Alongside the above-mentioned issues, other factors may contribute to improving the effectiveness of training programs, in terms of their transfer effects. Among such factors, control conditions [20], participant engagement [41, 42], and practice distribution [43, 44] have been found to modulate the effects of training. In the present study, we focus particularly on practice distribution to explore the relationship between trained and transfer tasks. The term *distributed practice* encompasses both *spacing effects* (i.e., the advantage of spaced over massed practice) and *lag effects* (i.e., the advantage of spacing with longer as opposed to shorter lags) [45]. Generally speaking, there is substantial evidence of spaced practice being superior to massed practice, and of performance benefiting more after a longer rather than a shorter lag ([46], for review).

In the context of WM training studies, however, evidence of the practice distribution effect is scarce and inconsistent. In the study by Penner et al. [44], two groups were trained using the BrainStim program, which included some WMU measures, over the course of 16 sessions. Performance in different cognitive domains was better in the training group that trained twice weekly (spaced distribution) than the performance shown by a group trained four times a week (massed distribution). A different pattern emerged in a study by Alloway et al.[43], in which a group trained four times a week for 8 weeks (massed distribution) outperformed another group trained with the same WM tasks only once a week for 8 weeks (spaced distribution), but this may have been due to the different amount of practice involved (one group attended 28 sessions, the other only 8). Hence the further aim of the present study was to assess potential practice distribution effects on the efficacy of WMU training programs. In line with the majority of studies on practice distribution, a superiority of the spaced practice groups was expected.

### The present study

The aim of this study was to examine the efficacy of two WMU programs for young adults. The mechanisms underlying transfer effects of WM training remain unclear, so we examined the conditions under which transfer occurs as well as some of the conditions that may facilitate it (see [22–24] for similar claims). We therefore newly examined the extent to which transfer is related to an improved updating process per se or to the acquisition of task-specific strategies applicable to structurally similar tasks. Very few published training studies have addressed this important issue.

Two training programs, based on two updating paradigms, were used, i.e. the *n*-back and arithmetical updating. As far as we know, this is the first study that focuses on the same process (WMU), and compares the transfer effects of two different WMU training programs. Performance in the training and transfer tasks was examined by administering various updating tasks as well as complex WM and reasoning tasks. The choice of transfer tasks stemmed from a theoretical approach to the categorization of mental processes. The trained and transfer updating tasks could be structurally similar (based on the same updating paradigm) or dissimilar (based on different updating paradigms).

The *n*-back and arithmetical updating tasks were chosen as training tasks because they tap the same process, i.e., WMU. Both types of updating task have been considered as indicators of the same latent factor [47]. Wilhelm et al. [48] reported a very high correlation (*r* = .92) between a latent factor consisting of *n*-back tasks and another factor comprising tasks similar to the arithmetical updating task, and they argued that both types of task measure the same updating construct. Both paradigms also involve constituent processes of WMU, such as information retrieval and substitution [49]. The *n*-back task is probably the most widely used in WMU training programs (e.g., [9–11]), and the arithmetical updating task has already been used in a previous training study and shown to produce specific training gains, at least [29].

Each of the two training programs included two training tasks to prevent fatigue and boredom, and promote engagement and motivation. Two versions of a single *n*-back task were used in the *n*-back training program; one involving numbers, the other letters. As for the arithmetical training program: one task involved participants applying different numerical operations to initial numbers that appeared in different boxes, and then recalling the last result obtained in each box [50, 51]; in the other task, they had to remember the lowest or highest number presented in each box, according to an initial criterion [52, 53].

An active control group was included in the study, as recommended by the literature (e.g., [20, 23]) to control for other experiment-related variables, such as contact with the experimenter or assessment context. This active control group played two casual video games used in previous training studies [15, 54–57], which do not specifically tap WM processes.

The efficacy of the training procedures was analyzed in terms of nearest transfer effects to four other WMU tasks that involved the same updating process as in the training, but differed from the trained tasks in terms of structural similarity: arithmetical updating; categorical updating; numerical *n*-back; and categorical *n*-back. There were two tasks for each of the two paradigms of interest (*n*-back and WM updating), and one of the tasks for each paradigm was structurally similar to the trained task, while the other was less so (see Table 1). This enabled us to examine transfer between updating paradigms, and nearest transfer to structurally similar tasks based on the same or different paradigms.

**Table 1:** Transfer tasks classified by type of transfer and degree of structural similarity, for each training paradigm.

		Arithmetical training	*N*-back training
Nearest transfer	Arithmetical updating task	Same task	Structurally dissimilar
	Categorical updating task	Structurally similar	Structurally dissimilar
	Numerical *n*-back task	Structurally dissimilar	Same task
	Categorical *n*-back task	Structurally dissimilar	Structurally similar
Near transfer	Operation span task	Different process	Different process
Far transfer	Cattell test	Different process	Different process

In the arithmetical training program, one transfer task was the same as a trained one (the criterion task: arithmetical updating), enabling us to measure specific effects in the group attending this training. Nearest transfer effects to structurally similar tasks were investigated by means of the categorical updating task, in which participants had to remember the last stimulus (an animal) that appeared in each box. These arithmetical and categorical WMU tasks shared some updating subprocesses that needed to be applied in the same order, including: focus switching and cued item information retrieval, item comparison, judgment about updating, and substitution of information. The other two WMU tasks (numerical *n*-back and categorical *n*-back) served to assess nearest transfer to tasks associated with the other updating training paradigm, and were therefore structurally dissimilar.

In the case of the *n*-back training program, one task (the criterion task: numerical *n*-back) mirrored the task practiced during the training, and elucidated specific effects in this training group. The categorical *n*-back task was included to assess nearest transfer to the trained task in a structurally similar task: participants had to indicate whether the word presented *n* steps before a target word corresponded to an animal. The numerical and categorical *n*-back tasks required a similar set of cognitive processes that had to be combined in a similar sequence: maintaining an active subset of *n* items in memory, making a recognition judgment to decide if the new item matched the first one in the subset, selecting a yes/no response, removing the first item in the subset, and adding the one presented more recently. The arithmetical updating task and categorical updating task were used to shed light on nearest transfer effects to the structurally dissimilar WMU tasks associated with the other updating training paradigm.

A classical WM task that measures the same narrow ability as the one trained, and a fluid intelligence test were also administered to assess near and far transfer effects, respectively. The inclusion of a reasoning measure allowed us to clarify if WMU training can produce–or not–far transfer effects, and depending on the results, thanks to the training tasks used, the possible mechanisms involved in presence or absence of transfer effects.

To explore the influence of practice distribution on transfer effects, two practice distribution schedules were used with the three groups of participants (the arithmetical and *n*-back training groups, and the active control group). This manipulation led to the inclusion of six groups in the present study. Within each training program, one group was trained using a massed distribution, and the other using a spaced distribution. Participants in the massed distribution groups attended all six training sessions during the same week, whereas the spaced distribution group attended training twice a week for three weeks.

Finally, self-perception and motivation were also taken into account, as there are initial evidences of their impact on training gains (i.e., [30, 42, 58, 59]). To give an example, Carretti et al. [60] found that motivational variables explained a certain amount of variance in training-related improvement when testing a program for older people. In young adults, Maraver et al. [59] likewise showed that strongly-motivated participants performed better in both trained and near transfer tasks. It might be argued that motivation can change the way participants approach training tasks. Greater motivation could be associated, for instance, with a greater engagement, a stronger commitment to improving performance, or a greater persistence after making a mistake [61]. Other authors identified no relationship between motivation or self-perception of improvement and performance, however. Zhao et al. [61] found that higher levels of motivation were related to greater training gains, but not to transfer effects. Fellman et al. [62] reported that participants’ self-perceived training gains were not substantiated by their actual performance. Given these inconclusive results, it seemed worthwhile to further investigate the link between motivational and self-perception variables on the one hand, and training-related performance on the other. Expectations, self-perceived improvement, and motivation were assessed at different times.

#### Study hypotheses

Various hypotheses derived from previous studies. If nearest transfer is mediated by the successful application of a strategy learned during the training to a similar transfer task, then performance gains will emerge for the updating tasks structurally similar to the tasks used in the training, but not for the tasks associated with the untrained paradigm. In other words, as concerns nearest transfer effects, the arithmetical training program would only prompt an improvement in the arithmetical updating task and in the categorical updating task; and the *n*-back training program would only yield a specific improvement in the numerical *n*-back task and in the categorical *n*-back task.

On the other hand, if nearest transfer effects are due to a strengthening of the updating process, performance gains will be seen across all WMU tasks, irrespective of the paradigm involved. In other words, both the arithmetical and the *n*-back training groups would show greater improvements than the active control group on all updating tasks.

Finally, as regards near and far transfer tasks, improvements in the operation span task and Cattell test would be expected only if and when the WMU process is enhanced (given the close relationship between WM and fluid intelligence (e.g. [63]), not if a strategy is acquired during the training, and no training effect should be seen in the active control group.

To assess the effect of practice distribution, we adopted a scheme similar to the one used in the study by Penner et al. [44], changing the lag between sessions and keeping the total number of sessions constant. We expected to find spaced superior to massed distribution, as in the Penner et al. [44] study (see [64], for other types of training).

As regards participants’ individual characteristics, their expectations, self-perceived improvement, and motivation were assessed at various times. These measures were included both to obtain participants’ feedback on how engaging they found the training, and to identify any relationship between motivation and training gains. Given the inconclusive results in the literature, we had no starting hypothesis on the relationship between motivation-related variables and performance on cognitive tasks.

## Method

### Participants

Participants were recruited from the University of Jaén, southern Spain and included 193 psychology and social work undergraduates (*M*_*age*_ = 21.36, *SD* = 3.49; 40 males, 153 females). After the pretest, participants were allocated to one of six study groups, based on practice distribution (massed or spaced), and type of training (arithmetical or *n*-back) or active control. They were allocated to a given group using a stratified random sampling method that took into account their pretest score in the Cattell test [65], and their university degree course (psychology or social work). Demographic details for each group are shown in Table 2. All participants were native Spanish speakers. No other inclusion/exclusion criteria were considered. The students volunteered for the study and received course credits for their participation.

**Table 2:** Demographic details of the sample, by type of training or active control, and by practice distribution.

Group		Age	Gender		Univ. degree		Cattell test
			Male	Female	Psychology	Social work	
Arithmetical	Massed	22.18 (6.52)	8	25	24	9	23.44 (4.61)
	Spaced	21 (21.19)	7	25	23	9	23.38 (3.77)
*N*-back	Massed	21.66 (2.88)	7	25	23	9	24.25 (4.51)
	Spaced	21.42 (2.19)	6	25	23	8	24.03 (3.66)
Act. control	Massed	20.52 (1.48)	6	25	23	8	23.39 (4.89)
	Spaced	21.32 (3.06)	6	28	24	10	24.47 (5.17)

*Note*. Standard deviations are shown in parentheses.

### Cognitive assessment

#### Criterion tasks

*Arithmetical updating task*. This task is similar to the one described by Oberauer et al. [50], and by Salthouse et al. [51], and was adapted by Linares et al. [29]. It was also used when training the arithmetical WMU group. Different lists of numbers and arithmetical operations were presented in different boxes. Participants had to memorize some numbers, apply different arithmetical operations, and remember the last result obtained. Each list started with a series of initial numbers inside their respective boxes. The number of boxes varied according to the memory load (i.e., the number of elements to remember in each list, which ranged from 1 to 5). After the initial numbers, nine arithmetical operations were presented, which involved subtraction or addition signs and the operands 0, 1, 2, 3. Each number or operation was displayed for 2500 ms with a 500 ms pause between items.

**Fig 1:**
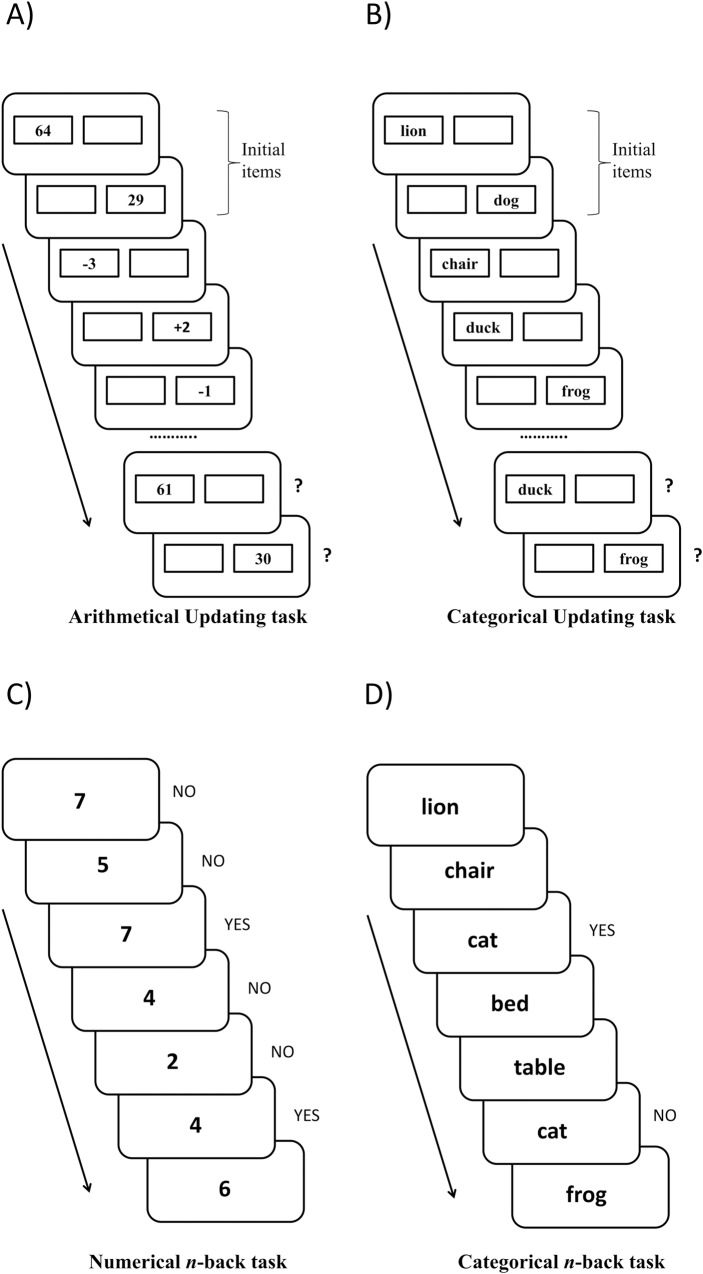
Nearest transfer tasks. The different panels show examples of incomplete sequences for each WMU nearest transfer task. Panel A shows the arithmetical updating task administered to the arithmetical training group, and panel B the untrained categorical updating task that shares the same structure as the arithmetical updating task. Panel C shows the numerical *n*-back task (at 2-back level) administered to the *n*-back training group, and panel D the 2-back level of the structurally similar untrained categorical *n*-back task.

A set of three lists for each memory load (1 to 5) was presented, starting with load 1. If participants completed two out of three lists correctly, the memory load increased in the next set. The task ended when participants failed in at least two of the three lists in the same set, or when they completed the set for load 5. No feedback was provided. Four practice lists were presented at the outset (load 1 and 2). The dependent variable was the percentage of correct answers (see panel A in Fig 1).

*Numerical n-back task*. This task was introduced by Kirchner [66] in 1958 and has since been widely used as a WMU measure. The same task was used to train the *n*-back WMU group. Different numbers from 1 to 9 were presented consecutively in the center of the screen and participants had to decide whether the number shown at a given moment corresponded to the one presented *n* positions previously. The numbers were each displayed for 1500 ms with a 500 ms pause between them.

Participants performed two 2-back followed by two 3-back lists. The memory load for each list depended on the number of items to be retained simultaneously in memory (2 for 2-back, and 3 for 3-back lists). A practice list for each level was administered before the test lists. Each list contained 32 items, namely 10 targets (numbers that matched those presented *n* positions earlier) and 22 non-targets (numbers that did not match). The dependent variable was a sensitivity index (d’) calculated by subtracting the z-score for the false alarms from the z-score for the hits (see panel C in Fig 1).

#### Nearest transfer tasks

*Categorical updating task*. This task was structurally similar to those used to train the arithmetical WMU group. Different lists comprising six animal and three object words were presented in different boxes. As with the training tasks, the number of boxes varied according to the memory load (the number of items to be memorized, which ranged from 2 to 5). In all lists, participants had to memorize the last animal word that appeared in each box and ignore the object words. Each word was displayed for 2000 ms with a 500 ms pause between items.

There were three lists for each memory load level (from 2 to 5). If participants completed two out of three lists correctly, the memory load increased in the next set. The task ended when participants failed in at least two lists for a given memory load. No feedback was provided. Four practice lists (load 1 and 2) were administered before the test lists. As with the training tasks, the dependent variable was the percentage of correct answers for each list (see panel B in Fig 1).

*Categorical n-back task* (adapted from Ciesielski et al. [67]). This task was structurally similar to those used to train the *n*-back WMU group. Different sequences including both animal and object nouns were presented consecutively in the center of the screen. Every time the animal word “GATO” (“cat” in English) appeared, participants had to indicate whether the noun presented *n* positions previously corresponded to an animal or an object (by pressing the J key for animals, and the F key for objects). They did not have to respond when an object word or an animal word other than GATO was presented. Each list contained a total of 40 words, included the word “GATO” that appeared 10 times, and required 5 responses of each type (object and animal). Each word was displayed for 1500 ms with a 500 ms pause between words.

Participants were administered two 2-back lists followed by two 3-back lists. A practice list for each level was administered prior to the test lists. The same dependent variables were considered as in the numerical *n*-back task (see panel D in Fig 1).

#### Near transfer task

O*peration span task* [68]. In this task, lists of paired arithmetical expressions (e.g., 2 + 4–1 = 5) and single-digit numbers (e.g., 6) were organized into sets of 2 to 7 items that were to be remembered. Half of the arithmetical expressions were correct. Each expression in a list was displayed for 5000 ms, and participants were asked indicate whether it was correct or not. This was followed by a blue number in the center of the screen for 3000 ms, which participants had to memorize. At the end of each list, participants had to recall all the blue numbers that had appeared after each arithmetical expression in the list, in the order of their presentation. There were two lists for each memory load level. The task was discontinued when a participant failed in both lists for the same memory load. Before starting the task, four practice lists were presented. The dependent variable was the percentage of correct answers.

#### Far transfer task

*Culture Fair Intelligence Test*, Scale 3 [65]. This scale from the Cattell test consists of four subtests. The first, *Series*, required that participants choose one of six options that best fitted an incomplete series of abstract shapes and figures. In the second subtest, *Classifications*, different drawings relating to five abstract shapes and figures were presented, and participants had to choose the two that differed from the other three. In the third subtest, *Matrices*, participants had to select one of six possible solutions that best completed several matrices containing from four to nine boxes containing abstract figures and shapes, plus one empty box. Finally, in the fourth subtest, *Conditions*, participants were presented with sets of abstract figures, lines and a single dot, along with five options. They had to assess the relationship between the dot, figures and lines, and choose the option in which the dot was in the same relationship with the other items. These subtests had to be completed within a set time, which ranged from 2.5 to 4 minutes, depending on the subtest. The dependent variable was the number of correctly solved items across the four subtests (maximum score 50).

#### Expectations, self-perceived improvement and motivation

Participants were asked a number of questions to examine the role of individual differences relating to their expectations, self-perceived improvement after the training, and motivation and strategy use during the training. At the beginning of the first session, participants were asked whether they expected their performance to improve. At the end of the training, they were asked about their self-perceived overall improvement in the trained tasks. They had to indicate the degree to which they thought they could improve, or had improved, on a scale of 1 to 10. Self-perceived improvement in each task was also examined using an online questionnaire that was sent to participants after the posttest session (see S1 Appendix).

As for motivation, participants were asked at the beginning of the first training session, after the third, and after the last, to indicate their level of motivation to complete the training program successfully on a scale from 1 to 10.

### Training activities

#### Arithmetical WMU training (massed and spaced groups)

*Arithmetical updating task*. For this task, 18 lists of initial numbers and arithmetical operations were presented. Each list started with various initial numbers, depending on the memory load (from 2 to 5 items to be memorized), each of which was displayed inside a box. Then a variable number (from 6 to 9) arithmetical operations (±0, ±1, ±2 or ±3) were displayed at random inside different boxes. The initial numbers were displayed for 2000 ms inside a box from left to right. Participants had to memorize the initial numbers for each box. Then they had to apply the arithmetical operation that appeared in each box to the corresponding number and memorize the result for that box. The next operation that appeared in the box had to be applied to the latest result for the box. At the end, participants had to type the last result memorized for each box. Feedback was provided after each list.

The level of difficulty changed, depending on the participants’ performance: when they completed two lists at the same difficulty level correctly, the memory load increased for the next list (meaning that they had to recall three items of information instead of two, for instance). If they failed for two consecutive lists, the memory load decreased by one item. The dependent variable was the mean memory load level correctly completed in each session.

*Number size updating task*. This task was based on Carretti et al. [52], and Lendínez et al. [53], and was adapted by Linares et al. [29]. Eighteen lists of numbers were displayed in different boxes. Participants had to memorize the last number in each box according to a given criterion: the smallest or largest number for each box. After presenting the criterion for a given list, the first numbers (ranging between 10 and 99) were presented consecutively, each in their own box, from left to right, for 2000 ms. New two-digit numbers were then displayed in different boxes chosen at random for 2000 ms. At the end of the list, silver boxes appeared and participants had to type the last number of each box that met the criterion. As with the previous task, feedback was provided.

This task included two types of item, updating and non-updating: the former met the criterion of their corresponding lists, while the latter did not. The memory load levels were the same as in the arithmetical updating task. The dependent variable was the mean memory load of each session completed successfully.

#### *N*-back WMU training (massed and spaced groups)

*Letter n-back task*. In this *n*-back task (adapted from Pelegrina et al. [6]), different letters of the alphabet were presented one at a time in the center of the screen. For each letter, participants had to decide whether it was the same as the letter presented *n* positions previously, pressing one key if the letters matched, or another key otherwise. This task thus involved controlling, maintaining and continuously updating the items presented throughout the sequence.

Twelve sequences of letters were presented at each session. Each sequence included 29 items requiring a response, one in three of which were target items (i.e., the letter presented was the same as the one presented *n* steps earlier). Each letter was displayed for up to 1500 ms or until participants gave their answer. The inter-stimulus interval was 1000 ms. Feedback was provided at the end of each sequence.

This task included different levels of difficulty, depending on the memory load (from 2 to 7 items to be memorized). Participants started the task by comparing the letter being displayed with the one presented 2 positions earlier. If they completed at least 80% of the sequence correctly, the memory load increased. From then on, the memory load decreased again if less than 60% of their answers were correct. The numbers of hits, false alarms, misses, correct rejections, and non-responses were recorded.

*Number n-back task*. This task was the same as the previous one but used numbers instead of letters. Twelve sequences of numbers from 1 to 9 were presented consecutively in the center of the screen and participants had to decide whether the number displayed matched the one presented *n* positions earlier. The same memory load levels and dependent variables were used as in the previous task.

#### Active control (massed and spaced groups)

*Bejeweled*. This task was similar to the popular casual game Candy Crush. Different gems were presented in an eight-by-eight grid. Participants had to swap adjacent identical gems to make sets of three. When four or more identical gems were swapped over, a power gem scoring extra points was created for the next swap. The level of difficulty increased as participants earned more points. An endless version of the task was used, and the task was terminated after 15 minutes. The dependent variable was the game score achieved at the end of the session.

*Dodge the Meteor*. This task involved participants moving a spacecraft across the screen using the arrow keys to reach different objects (e.g., a hamburger) briefly appearing on the screen. In addition to the spacecraft and objects, a number of meteors that could hit the spacecraft moved across the screen. Participants had to dodge the meteors and shoot them several times to make them disappear. The dependent variable was the game score achieved at the end of the session.

### Procedure

Participants were recruited on a voluntary basis from different university courses in psychology and social work studies and received course credits for their participation. They were informed that they had the opportunity to participate in a training study involving memory tasks and video games that had reportedly produced some performance gains in other study populations.

Participants attended eight sessions. The first and eighth sessions were for assessment purposes, and the remainder were training sessions. During the assessment sessions, tasks were administered in the order described in the Materials section. Two parallel versions (A and B) were constructed for each task, and counterbalanced across all assessment sessions. The pretest session took place the day before starting the training, and the posttest session at least one day after completing the training. The assessment sessions lasted an hour.

Each of the six training sessions lasted approximately 30 minutes, during which each training group performed two tasks, administered in the order described in the Materials section. Six study groups were formed by combining the two types of training (arithmetical training, *n*-back training) and the active control activity with two different practice schedules: massed and spaced. In the massed practice groups, participants attended all training sessions in the same week, with no more than two sessions on the same day. In the spaced practice groups, they attended two sessions a week, with in interval of at least one day between sessions.

All assessment and training tasks were administered individually in booths. During the assessment sessions, the experimenters stayed with participants to explain each task to them, while participants were left alone in the booths during the training sessions. The experimenters and participants were aware that there were different types of training involved, but they were not informed about the aims of the study.

The ethics committee at the University of Jaén approved the study. All participants gave their written informed consent before taking part. After completing the study, all participants had the opportunity to receive information about the objectives and outcome of the training program.

## Results

The analyses of the results are presented in four parts. First, we investigated differences between the groups at pretest. Then we analyzed standardized performance gains for each group during the training. Third, we examined gains from pretest to posttest on the assessment tasks, and compared their corresponding effect sizes. Finally, we explored possible differences in training and transfer task performance relating to participants’ levels of engagement and motivation.

### Baseline measures

To ensure that the six groups did not differ on any of the tasks at pretest, separate 3 (Group: arithmetical, *n*-back, active control) x 2 (Practice distribution: massed, spaced) unifactorial ANOVAs were run on pretest performance across all measures. The results revealed no between-group differences at pretest for any of the measures of interest (*p* > .369).

### Training gains

Training gains were assessed by taking into account the mean level reached for each task at each session. A 6 (Sessions 1 to 6) x 2 (Practice distribution: massed, spaced) mixed-design ANOVA was run for the mean level reached in each task, with practice distribution as the between-subjects factor and sessions as a repeated measure.

#### Arithmetical training group

**Fig 2:**
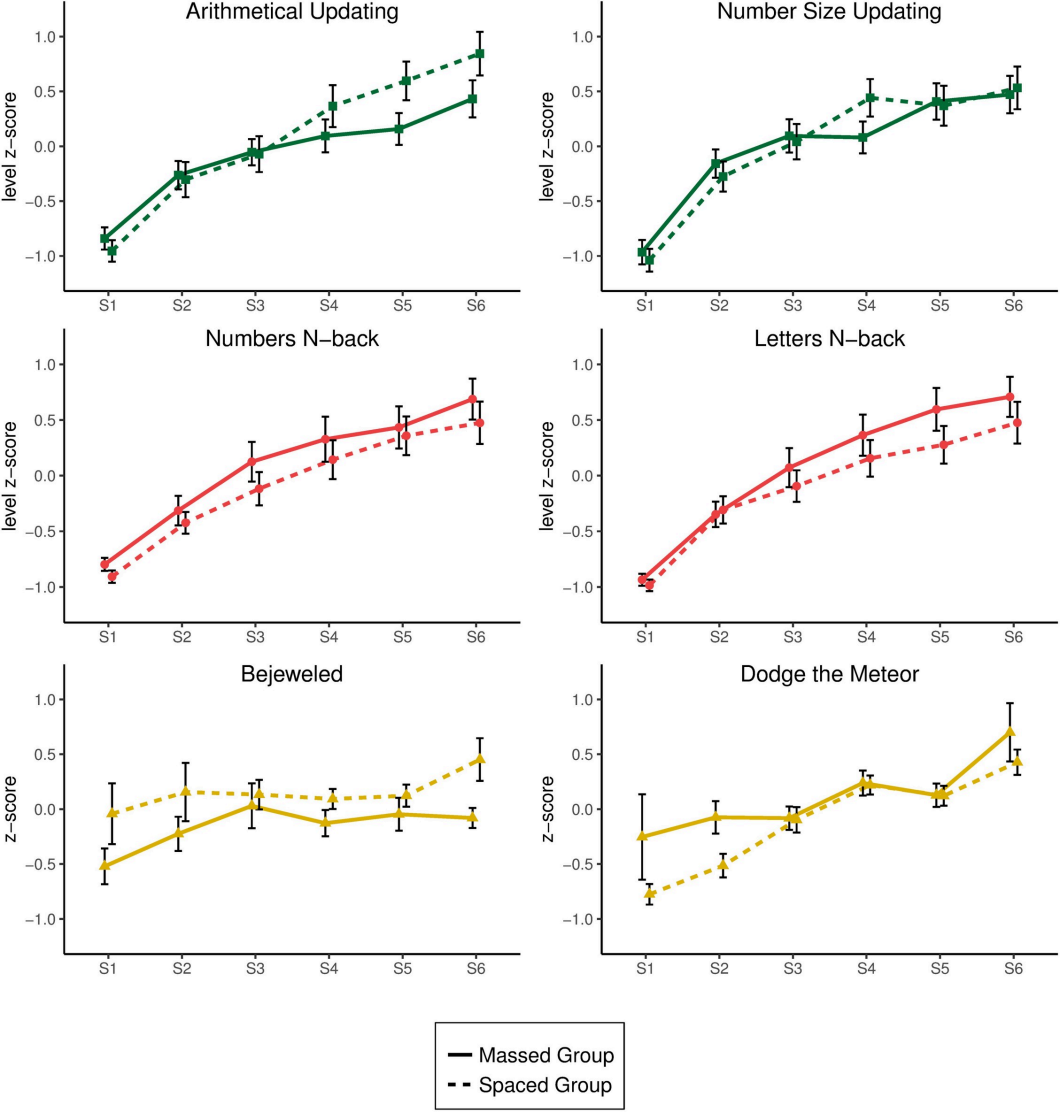
Mean level reached in all tasks across the six training sessions for the arithmetical, *n*-back, and active control groups. The bars represent two standard errors of the mean.

An effect of session was found for the arithmetical updating task, *F*(5, 310) = 72.64, *p* < .001, ŋ^2^_p_ = .54, and the interaction between session and practice distribution was significant, *F*(5, 310) = 3.66, *p* = .003, ŋ^2^_p_ = .06. This was explained by the interaction between the linear trend for session and practice distribution, *F*(1, 62) = 8.96, *p* = .004, ŋ^2^_p_ = .13. This interaction showed a slightly more pronounced improvement in performance after training with a spaced practice (Session 6 –Session 1) than after a massed practice (*Mdiff* = 1.80; *Mdiff* = 1.27, respectively). The main effect of practice distribution was not significant (*p* = .389) (see top left panel in Fig 2).

The effect of session was significant for the number size updating task too, *F*(5, 310) = 57.87, *p* < .001, ŋ^2^_p_ = .48. Analyzing the trend, the linear component reached significance, *F*(1, 62) = 144.17, *p* < .001, ŋ^2^_p_ = .70, accounting for 84.95% of the variance. The quadratic component, *F*(1, 62) = 31.03, *p* < .001, ŋ^2^_p_ = .33, and the cubic component, *F*(1, 62) = 7.82, *p* = .007, ŋ^2^_p_ = .11, were significant as well, explaining 12.42% and 2.29% of the variance, respectively. Neither the interaction between session and practice distribution nor the main effect of practice distribution reached significance (*p* > .199) (see top right panel in Fig 2).

#### *N*-back training group

The mean level reached for each task in each training session was also used to assess training gains for the *n*-back training group.

For the numerical *n*-back task, the effect of session was significant, *F*(5, 305) = 83.89, *p* < .001, ŋ^2^_p_ = .58. A trend analysis revealed a significant linear component for session, *F*(1, 61) = 145.73, *p* < .001, ŋ^2^_p_ = .70, which explained 95.02% of the variance. The quadratic component, *F*(1, 61) = 16.44, *p* < .001, ŋ^2^_p_ = .21, also reached significance, accounting for 4.62% of the variance. The interaction between session and practice distribution and the main effect of practice distribution were not significant (*p* > .428) (see middle left panel in Fig 2).

A significant main effect of session was found for the letter *n*-back task too, *F*(5, 305) = 86.26, *p* < .001, ŋ^2^_p_ = .59. The trend analysis revealed that the linear component, *F*(1, 61) = 140.98, *p* < .001, ŋ^2^_p_ = .70, the quadratic component, *F*(1, 61) = 22.04, *p* < .001, ŋ^2^_p_ = .27, and the cubic component, *F*(1, 61) = 7.51, *p* = .008, ŋ^2^_p_ = .11, were all significant, accounting for 92.97%, 6.30% and 0.60% of the variance, respectively. The interaction between session and practice distribution and the main effect of practice distribution did not reach significance (*p* > .356) (see middle right panel in Fig 2).

#### Active control group

In the Bejeweled game, the effect of session was not significant (*p* = .087), but the linear trend was, *F*(1, 62) = 5.71, *p* = .020, ŋ^2^_p_ = .08, and explained 69.91% of the variance. The main effect of practice distribution was also significant, *F*(1, 62) = 5.26, *p* = .025, ŋ^2^_p_ = .08, since performance after spaced practice was higher than after massed practice (*Mdiff* = 0.314). The interaction between session and practice distribution was not significant (*p* > .697) (see bottom left panel in Fig 2).

For the Dodge the Meteor game, the main effect of session was significant, *F*(5, 310) = 13.26, *p* < .001, ŋ^2^_p_ = .18. The trend analysis revealed a significant linear component for session, *F*(1, 62) = 41.10, *p* < .001, ŋ^2^ = .40, which explained 93.04% of the variance. Neither the main effect of practice distribution nor the interaction between session and practice distribution were significant (*p* > .112) (see bottom right panel in Fig 2).

#### Transfer effects

Some participants were excluded from the analyses on transfer effects for the following reasons: 1) two participants were disregarded because one of them only attended the pretest session, and the other did not complete the pretest session due to eyesight problems; 2) two participants did not complete the arithmetical updating task at pretest, and were consequently not considered in the analysis of this task; 3) one participant was excluded from the analysis of the Cattell task at pretest because the timing of its administration was not properly monitored; 4) the data files pertaining to one participant’s performance in the categorical updating task, the categorical *n*-back task, and the operation span task at pretest were inadvertently overwritten, so they could not be included in the analyses; and 5) two participants’ performance in the arithmetical updating task, and three participants’ performance in the categorical *n*-back task were not analyzed due to their extremely low scores (mean accuracy below 10%), from which we inferred that they had not understood the task in the pretest session.

To ascertain the effect of training, performance gains in each task were calculated as the difference between the pretest and posttest scores. Performance gains for each group were then compared by running three sets of 2 (Group) x 2 (Practice distribution) ANOVAs on the gains for each task, with groups and practice distribution as between-subjects factors. The results are summarized in Table 3. The same results are obtained using z-scores.

**Table 3:** Results of three sets of two-way ANOVAs for the measures of interest, with group and practice distribution as between-subjects factors.

		Arithmetical training vs. Active control				*N*-back training vs. Active control				Arithmetical training vs. *n*-back training			
Transfer task		*F*	*df*	*p*	ŋ^2^_p_	*F*	*Df*	*p*	ŋ^2^_p_	*F*	*df*	*p*	ŋ^2^_p_
WMU arithmetical task	G	29.43	1, 121	< .001	.20	1.45	1, 120	.230	.01	20.05	1, 121	< .001	.14
	PD	1.30	1, 121	.256	.01	0.27	1, 120	.606	.00	1.26	1, 121	.264	.01
	G x PD	0.44	1, 121	.507	.00	0.00	1, 120	.964	.00	0.51	1, 121	.476	.00
WMU categorical updating	G	5.45	1, 123	.021	.04	0.04	1, 122	.844	.00	4.55	1, 123	.035	.04
	PD	1.67	1, 123	.199	.01	1.30	1, 122	.257	.01	0.55	1, 123	.459	.00
	G x PD	0.04	1, 123	.850	.00	0.42	1, 122	.521	.00	0.12	1, 123	.735	.00
WMU numerical *n*-back	G	0.95	1, 124	.330	.01	43.18	1, 123	< .001	.26	42.21	1, 123	< .001	.26
	PD	0.62	1, 124	.431	.01	1.48	1, 123	.226	.01	3.94	1, 123	.049	.03
	G x PD	0.33	1, 124	.565	.00	1.05	1, 123	.308	.01	0.33	1, 123	.566	.00
WMU categorical *n*-back	G	0.90	1, 121	.344	.01	0.12	1, 120	.734	.00	1.51	1, 121	.221	.01
	PD	1.85	1, 121	.177	.02	0.03	1, 120	.869	.00	0.03	1, 121	.865	.00
	G x PD	0.13	1, 121	.714	.00	1.12	1, 120	.292	.01	2.04	1, 121	.156	.02
WM operation span	G	2.16	1, 123	.144	.02	0.99	1, 122	.321	.01	0.20	1, 123	.655	.00
	PD	0.05	1, 123	.817	.00	1.13	1, 122	.289	.01	0.91	1, 123	.341	.01
	G x PD	0.00	1, 123	.974	.00	1.60	1, 122	.209	.01	1.44	1, 123	.232	.01
Fluid intelligence (Cattell)	G	0.12	1, 123	.730	.00	1.76	1, 123	.187	.01	2.14	1, 122	.146	.02
	PD	1.61	1, 123	.207	.01	2.70	1, 123	.103	.02	1.35	1, 122	.247	.01
	G x PD	0.06	1, 123	.804	.00	0.00	1, 123	.953	.00	0.04	1, 222	.852	.00

*Note*. G = group; PD = practice distribution

#### Nearest transfer

**Fig 3:**
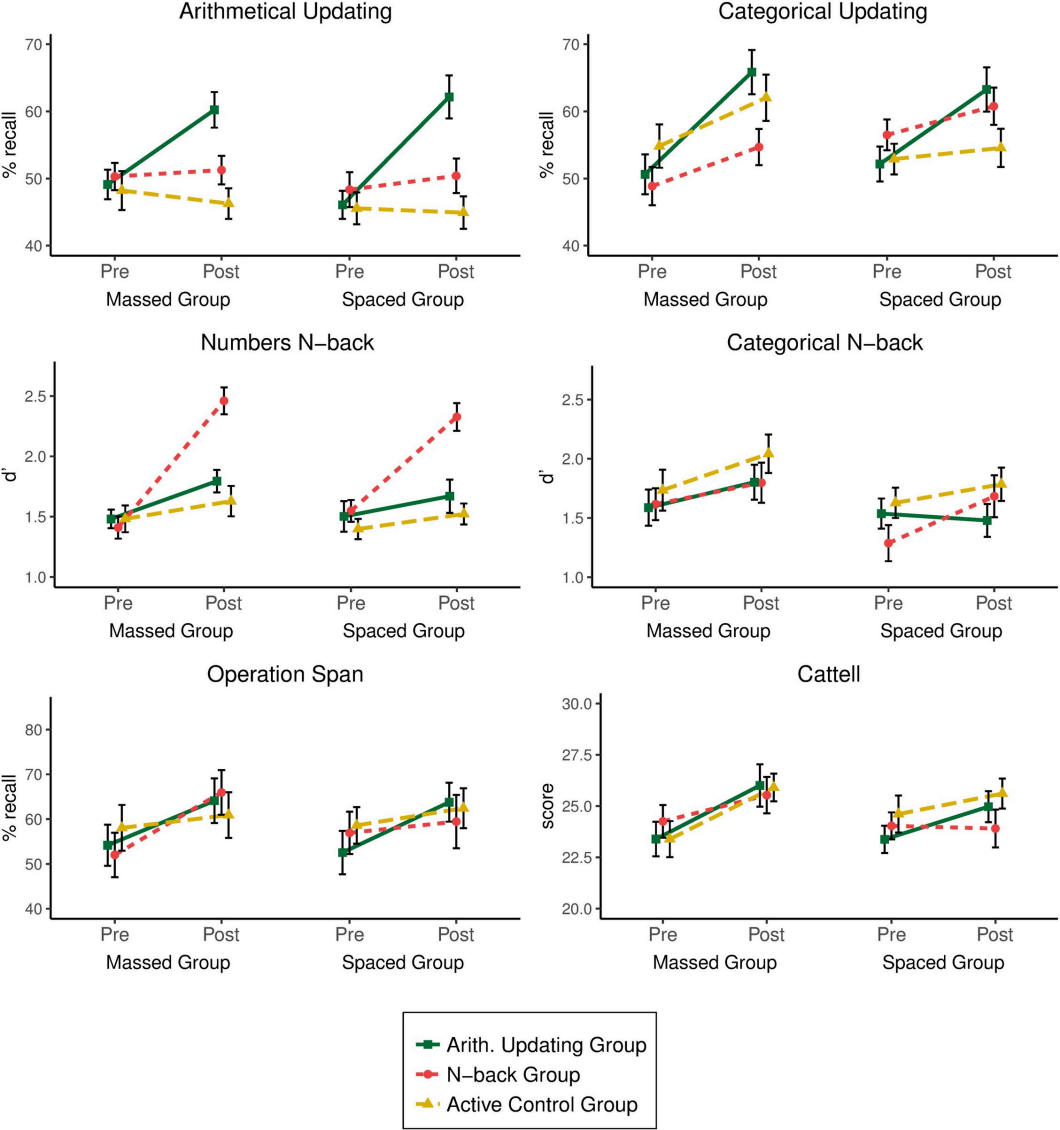
Scores obtained in all transfer tasks by group (arithmetical, *n*-back, active control) and practice distribution (massed, spaced).

For the arithmetical updating task, the arithmetical training group showed larger performance gains (*Mdiff* = 13.59) than the *n*-back training group (*Mdiff* = 1.50) or the active control group (*Mdiff* = -1.29). The gain in the *n*-back training group did not differ significantly from that of the active control group. Neither the main effect of practice distribution nor the interaction were significant (see top left panel in Fig 3).

In the numerical *n*-back task, performance gains were significantly greater for the *n*-back training group (*Mdiff* = 0.92) than for the arithmetical training group (*Mdiff* = 0.24) or the active control group (*Mdiff* = 0.14), with no differences in performance gains between the two latter groups. The practice distribution effect was significant in the comparison between the arithmetical and the *n*-back training groups, massed practice being associated with larger gains (*Mdiff* = 0.68) than spaced practice (*Mdiff* = 0.48). No other effects of practice distribution nor the interaction were significant (see middle left panel in Fig 3).

For the categorical updating task, the arithmetical training group showed larger gains (*Mdiff* = 13.16) than the *n*-back training group (*Mdiff* = 5.04) or the active control group (*Mdiff* = 4.43). Performance gains for the latter two groups did not differ. Neither the practice distribution effect nor the interaction were significant (see top right panel of Fig 3).

Finally, the analysis of performance in the categorical *n*-back task showed no differences in the gains made by any of the groups (arithmetical training: *Mdiff* = 0.08, *n*-back training: *Mdiff* = 0.29, and active control: *Mdiff* = 0.23). Neither the interaction nor the practice distribution effect reached significance (see middle right panel in Fig 3).

#### Near transfer

For the operation span task, there were no differences in the groups’ performance gains (arithmetical training: *Mdiff* = 10.60, *n*-back training: *Mdiff* = 8.23, and active control: *Mdiff* = 3.35). Neither the practice distribution effect nor the interaction proved significant (see bottom left panel in Fig 3).

#### Far transfer

The groups’ performance gains in the Cattell test did not differ statistically (arithmetical training: *Mdiff* = 2.10, *n*-back training: *Mdiff* = 0.58, and active control: *Mdiff* = 1.76). The practice distribution effect and the interaction also lacked significance (see bottom right panel of Fig 3).

**Fig 4:**
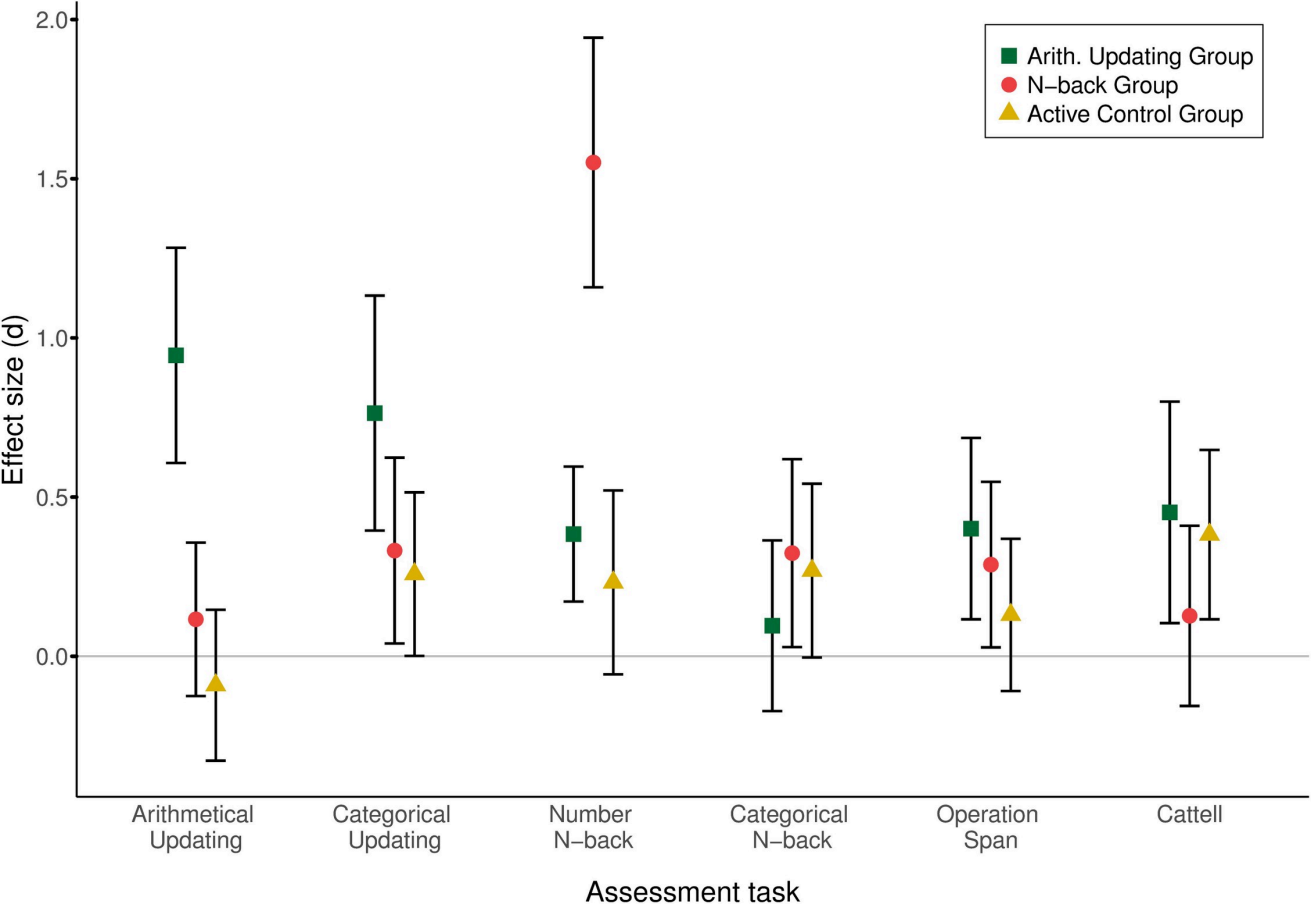
Effect sizes for each assessment transfer task by group (arithmetical, *n*-back, active control).

Cohen’s *d* [69] was calculated to compare the effect sizes at pretest and posttest for each assessment task administered to the three groups. Values higher than 0.80 are considered large effects. Geoff Cumming's ESCI Excel macro was used to calculate the confidence intervals around the effect sizes [70]. As shown in Fig 4, the arithmetical updating group showed a large effect for the same trained task, and an effect higher than 0.75 for the categorical updating task. The *n*-back group only showed a large effect for the same trained task. In addition to these large effects, there were small or moderate effects in almost all tasks, irrespective of the group.

### Individual characteristics

#### Engagement

All participants were interviewed about their expectations concerning any improvement in their performance before the training commenced. A 3 (Group: arithmetical, *n*-back, active control) x 2 (Practice distribution: massed, spaced) ANOVA was run on these measures. No differences emerged between the various groups (*p* = .159), and practice distributions (*p* = .471) at the start of the training. Overall self-perceived improvement was assessed at the last training session: there were no statistically significant differences between the groups (*p* = .138), but the practice distribution effect neared significance, *F*(1, 185) = 3.49, *p* = .063, ŋ^2^_p_ = .02. A look at the means indicated that overall self-perceived improvement tended to be somewhat higher for the massed practice (*M* = 7.12) than for the spaced practice (*M* = 6.70).

After the training, 93.78% of participants completed a final online questionnaire about their self-perceived improvement in each assessment task. A 3 (Group: arithmetical, *n*-back, active control) x 2 (Practice distribution: massed, spaced) ANOVA showed a significant main effect of group for all tasks: arithmetical updating, *F*(2, 114) = 16.55, *p* < .001, ŋ^2^_p_ = .22; numerical *n*-back, *F*(2, 114) = 20.46, *p* < .001, ŋ^2^_p_ = .26; categorical updating, *F*(2, 114) = 17.44, *p* < .001, ŋ^2^_p_ = .23; categorical *n*-back, *F*(2, 114) = 7.56, *p* = .001, ŋ^2^_p_ = .12; operation span , *F*(2, 114) = 8.26, *p* < .001, ŋ^2^_p_ = .13; and the Cattell test, *F*(2, 114) = 6.51, *p* = .002, ŋ^2^_p_ = .103, *F*(2, 114) = 6.51, *p* = .002, ŋ^2^_p_ = .10.

Post-hoc pairwise comparisons were performed with Bonferroni’s correction for multiple comparisons to see which groups differed from each other on self-perceived improvement in each task. As shown in Table 4, the *n*-back and arithmetical training groups scored higher on the assessment tasks associated with the same paradigm as the tasks used in their training. In the other tasks, the *n*-back and arithmetical training groups did not differ significantly. The active control group obtained lower scores than the training groups across all tasks. Neither the practice distribution effect nor any interaction between practice distribution and group were significant on any task (*p* > .166). In short, compared to the active control group, both training groups perceived that they had improved in the majority of tasks, and especially in the trained tasks.

**Table 4:** Means and standard errors for self-perceived improvement in each task by group.

	Group			
Assessment task	Arithmetical	*N*-back	Active control	Group comparisons
Arithmetical updating	5.64 (0.22)	4.87 (0.21)	3.77 (0.24)	Ar > Nb > Ac
Numerical *n*-back	4.67 (0.22)	5.42 (0.21)	3.39 (0.24)	Nb > Ar > Ac
Categorical updating	5.38 (0.23)	5.20 (0.21)	3.58 (0.24)	Nb = Ar > Ac
Categorical *n*-back	4.54 (0.23)	4.70 (0.22)	3.49 (0.25)	Nb = Ar > Ac
Operation span	5.16 (0.24)	4.80 (0.23)	3.75 (0.26)	Nb = Ar > Ac
Cattell	4.85 (0.23)	4.30 (0.21)	3.65 (0.24)	Ar > Ac, Ar = Nb = Ac

*Note*. Standard errors are shown in parentheses. Ac: active control group, Ar: arithmetical training group, Nb: *n*-back training group

#### Motivation

Motivation was assessed at the beginning, at the middle and at the end of the training. A 3 (Group: arithmetical, *n*-back, active control) x 2 (Practice distribution: massed, spaced) x 3 (Time: beginning, middle, end) mixed-design ANOVA was run on the level of participants’ motivation.

**Fig 5:**
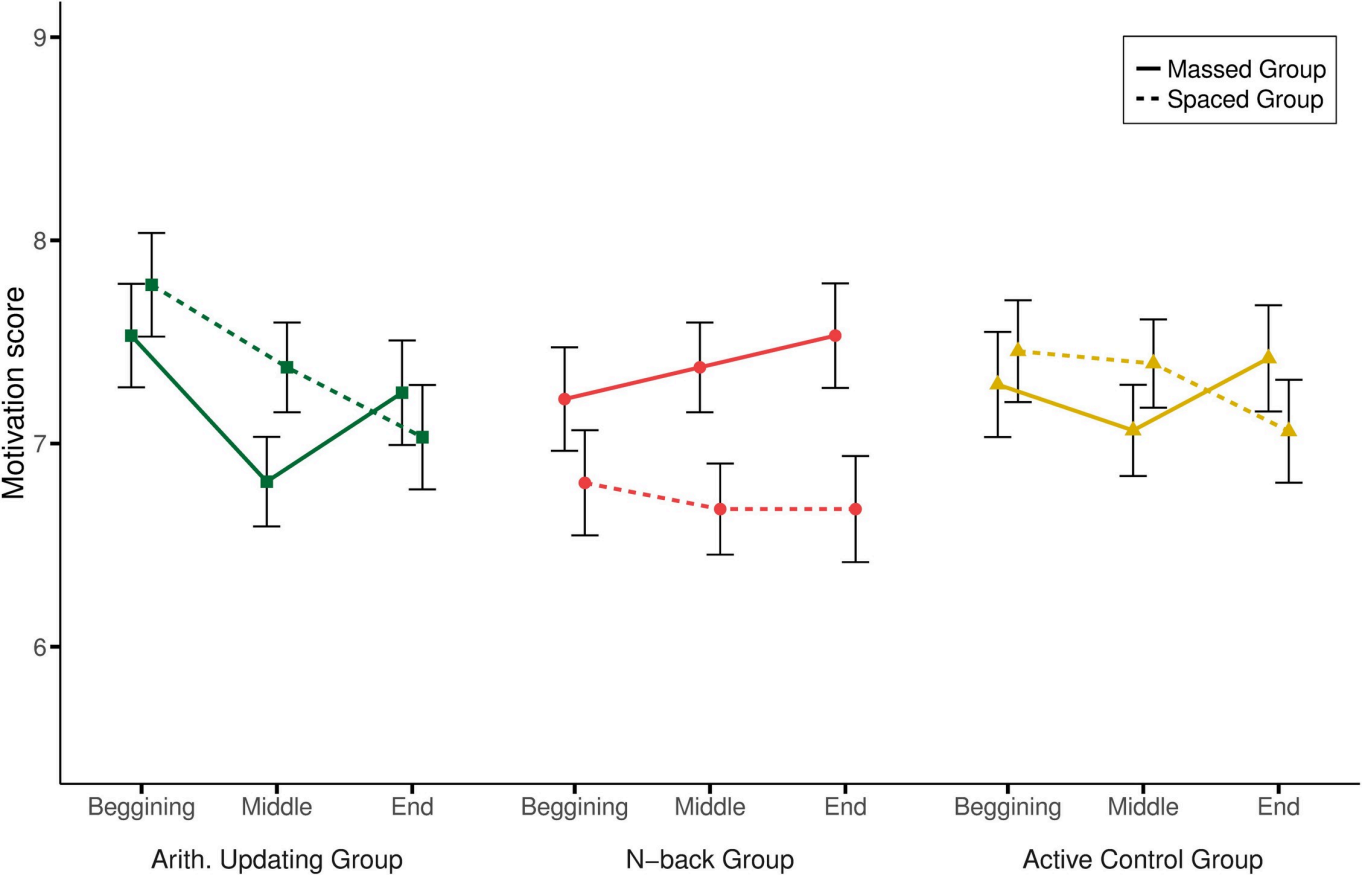
Level of motivation across the training (beginning, middle and end) by group and practice distribution.

Only the interaction between session and practice distribution was significant, *F*(2, 370) = 4.35, *p* = .014, ŋ^2^_p_ = .02. To clarify this interaction, the level of motivation was analyzed for each type of practice distribution. For massed practice, the effect of session was significant, *F*(2, 188) = 3.17, *p* = .044, ŋ^2^_p_ = .03, due to a greater motivation from the middle of training to the end (*Mdiff* = 0.32, *p* = .028). For spaced practice, the significant effect of session, *F*(2, 190) = 4.07, *p* = .019, ŋ^2^_p_ = .04, revealed a decline in motivation from the beginning to the end of the training (*Mdiff* = 0.43, *p* = .038) (see Fig 5). In short, a greater motivation coincided with the massed practice, while motivation decreased when spaced practice was used.

Taken together, differences in participants’ engagement and self-perceived improvement did not relate to their performance in the present study: the massed practice groups were reportedly more motivated, but they did not outperform the spaced practice group.

## Discussion

It is important in cognitive training research to shed light on the mechanisms behind transfer training effects (see [22–24]). A novel aspect of the present study lies in that we trained the same cognitive process using two different WMU training paradigms: *n*-back and arithmetical updating. We examined the extent to which the benefits of training in one paradigm transferred to other structurally similar tasks demanding the same updating process, to other structurally dissimilar tasks demanding the same updating process, and to other WM and reasoning measures. This enabled us to address two possible mechanisms involved in transfer effects: the strengthening of a common process, and the acquisition of strategies appropriate for structurally similar tasks.

### Transfer effects

#### Nearest transfer

Performance improved with training for all WMU training groups, and also for the active control group, indicating that the training was effective, and that all study participants took an active interest.

Nearest transfer was considered on two levels: between different updating paradigms; and to structurally similar tasks based on the same paradigm. There was no evidence of nearest transfer between the two updating paradigms used to train participants in the present study. The group given arithmetical updating training did not improve specifically in the *n*-back tasks, and the group given *n*-back training did not improve in the arithmetical or categorical updating tasks. These results may come down to the fact that, although the two types of paradigm share an updating requirement, they vary in other processes. For example, they may differ in terms of the retrieval operations (recognition vs. recall), and in how the item to be updated is selected (external or internal cues), and thus on the general strategy or approach used by the participant. Our results using two updating paradigms are therefore compatible with reports that performance improvements on one WM measure do not lead to gains on another type of WM task (e.g., [9, 11, 17]).

While nearest transfer effects between different updating paradigms proved elusive, when we looked for such effects on structurally similar tasks involving the same paradigm our results differed, depending on the updating paradigm. The arithmetical training group improved not only in the same trained task, but also in the structurally similar categorical updating task. In both tasks, an item presented in a box was an indication that it could be updated, and answers were given by inputting the last item in each box at the end of a list. The differences between the tasks were superficial, involving the type of material (numbers vs. letters), and the operations required (arithmetical operations vs. semantic judgments). As for the *n*-back training group, gains were only evident in the trained numerical *n*-back task, not in the similar categorical *n*-back task. These two tasks share many features that led us to consider them as structurally similar: they are *n*-back tasks that involve comparing a given item with one presented *n* positions earlier, meaning that the information retained for the previous position has to be retrieved and then substituted. There were some differences in certain superficial structural features, however, that might justify our result, including the type of material (numbers vs. letters), the type of operation (identity comparison vs. categorical judgment), and the response mode (yes/no to all words vs. only when a key word is presented). Our findings thus indicate that transfer to another, structurally similar updating task tapping the same process as the trained one [48] may sometimes prove unsuccessful.

This raises the important issue of how to decide which tasks are structurally similar. In the present study, similar tasks shared the same presentation and could be solved following similar procedural steps, whereas they differed in superficial features, such as the operations to perform (semantic vs. arithmetical), or the material to be remembered (words vs. numbers). Different criteria may have been adopted to define the structural similarity of tasks in other studies. Laine et al. [40] showed nearest transfer between very similar tasks, when they varied their trained and transfer *n*-back tasks only on content (letters and colors instead of numbers). Harrison et al. [71] used structurally similar training and transfer WM tasks that differed in both material and distractors. In short, it is still not clear when a feature of a task is superficial and irrelevant for the purposes of transfer effects, or whether there is a measure of structural similarity between trained and transfer tasks. Further training studies could experimentally manipulate the features of tasks to ascertain their relevance to transfer and thus determine the structural similarity of the tasks in question.

#### Near and far transfer

No near or far transfer effects came to light in the present study. Performance gains in a classical complex operation span, and a reasoning task (the Cattell test) were much the same among groups. Since the Cattell is a timed task, any general improvements in this measure may be due to participants becoming faster. Our results reflect those of other studies reporting no transfer effects to complex WM tasks [8, 9, 11, 27]. A lack of transfer effects from WMU training to fluid intelligence has been reported in a number of studies too (e.g., [14–17, 27]), and some meta-analyses have addressed this issue. For example, Melby-Lervag et al. [22] claimed that there is no evidence to support the notion that WM training improves performance in far-related abilities. A recent review on cognitive training [23] also emphasized that the strongest evidence for post-training gains tends to be in favor of nearest transfer, while broader transfer is considerably less frequent. Another issue that remains to be clarified is why some studies found far transfer effects–especially those related to fluid intelligence–even though no near transfer gains were apparent.

#### Why are transfer effects elusive?

To summarize, the present study found specific training gains, while there was nearest transfer only to a structurally similar WMU task involving one updating paradigm (arithmetical updating), not the other (*n*-back). Some explanations have been suggested for specific transfer effects (such as those observed in this study), which would be due to knowledge gained from the training (e.g., [72]) and to familiarity with the task and computer-based assessment methods. Such performance gains are also likely to be the result of task-specific strategies being applied, rather than any general improvement in a process [21].

Strategy use may have a crucial role when it comes to nearest transfer. Laine et al. [40] demonstrated that teaching participants a strategy improved their performance in trained tasks (*n*-back with digits), and in other structurally similar untrained tasks (*n*-back with letters and colors), but not in other, structurally dissimilar tasks (e.g., running memory span). The pattern of transfer to structurally similar tasks in the Laine et al. study [40] is comparable with the one seen here for arithmetical updating training, but not with our findings with the *n*-back training, since there was no evidence of transfer to the structurally similar task in the latter case. Thus, it is only in some circumstances that nearest transfer effects would emerge in tasks tapping the same process, and suitable for being solved using the same specific strategy as the one used in the trained task.

A strategy used for one trained paradigm might not be effective for another involving different processes. De Simoni and von Bastian [38] recently found that, even when participants reported using a similar strategy (e.g., rehearsal) for both a trained (e.g., memory updating) and a transfer (e.g., associative binding) task, performance gains were only seen for the paradigm used in the training, not for another paradigm.

Transfer effects may depend on the decision to use a strategy and on how successfully a learned strategy is applied. There are a number of factors involved in the decision to use a particular strategy over others, and factors associated with the actual implementation of a previously-learned strategy (see [73]). As regards the former, differences in some salient features, and in the configuration of transfer and training tasks may lead to different strategies being applied. Subjectively-perceived similarities between transfer and training tasks may influence the use of a strategy as much as objective similarities.

The processing requirements of a task may also influence the decision to apply a particular strategy. In other settings, it has been demonstrated that individuals use different strategies to perform the same tasks, and they tend to choose more complex strategies as they gain experience with them [74]. Asking participants about their strategies might shed light on this issue, unfortunately we did not collect information about that. Looking at the results it seemed that, our participants have developed a specific strategy (or a set of strategies) for updating information (as demonstrated from the increase during the training), but specific for the trained *n*-back task (as suggested from the lack of transfer). In other words, the learnt strategy was not adapted to the new task, although it made similar requests with apparently slight variations.

Factors related to the more or less successful use of newly-acquired strategies are also worth considering [73]. Transfer may be undermined when a new task involves an additional processing step, or requires a different type of response. In the present study, the categorical *n*-back included a semantic judgment that was not present in the trained *n*-back task. In addition, the training *n*-back task required an answer every time an item was presented, whereas a response was only needed in the transfer task when a keyword appeared. This may imply that the latter task included a processing component related to prospective memory. It may also require an additional inhibitory process as participants may have needed to refrain from responding to some items. Such additional processes may have hampered participants’ successful use of their previously-learned strategy.

The involvement of metacognitive knowledge may have had a part to play in strategy use too. For example, participants who showed no improvement in some tasks, such as the categorical *n*-back, may not have realized that the previously practiced strategy was applicable to the new task too. Such limitations would be similar to the production or utilization deficiencies seen in children in studies on memory strategy development [75].

### Practice distribution

The present study also aimed to explore the influence of a massed versus spaced practice distribution. Few studies have analyzed practice distribution in WM training programs, yielding different conclusions (see [40, 41, 64]). Our results also failed to reveal a clear pattern. On the one hand, spaced practice during the training was more effective than massed practice in two of six tasks (arithmetical updating and the Bejeweled game). These results are partially consistent with the superiority of spaced practice [44, 64]. Longer intervals between sessions might give participants with more opportunities to consolidate or re-acquire a strategy [46, 64]. The advantage for the spaced groups during the training only applied to two tasks, however, and there was no effect on transfer task performance. Judging from our present findings, massed vis-à-vis spaced practice distributions do not produce entirely consistent effects on transfer performance. We cannot offer a conclusive explanation for this pattern.

Practice distribution may influence motivation during the period of training, however, since our participants’ level of motivation increased with massed practice and decreased with spaced practice. Fellman et al. [62] also reported a significant decrease in motivation during the training. We speculate that motivation declines over time. Our training program with a spaced practice distribution lasted twice as long as the one involving massed practice, so participants attending the former should be more affected, in line with the classic report from Baddeley and Longman [76]. That said, it is worth mentioning that differences in levels of motivation had no impact on performance: the massed practice group did not outperform the spaced practice group. As for motivation, future studies should make the effort to match the groups at baseline on such a variable to cast light on its impact on training gains. The influence of practice distribution on motivation may warrant further investigation.

### Strengths and limitations of the present study

A novel feature of this study lies in that it explored the mechanisms underlying transfer of training benefits by administering of two different training programs that focused on the same updating process but based on different paradigms. To our knowledge at least, no studies had previously addressed such an issue, as either the same process was trained employing a single paradigm as the *n*-back (e.g., [8, 14]), or different paradigms that trained different processes (e.g., [10, 38, 77]). Our study also specifically considered nearest as well as far transfer, an issue that has become relevant in the light of recent studies (e.g., [11, 29]), and highlighted by some meta-analyses [22–24].

The programs in the present study consisted of a fairly small number of six sessions, but a meta-analysis confirmed that the number of sessions did not affect the effectiveness of a training based on *n*-back tasks similar to those used here [24]. Moreover, the effects of cognitive training are expected to be rather limited, so it would not be ethically appropriate to have participants training over many sessions without clear expectations of gains.

In conclusion, although previous studies have shown that transfer effects may depend on the variance shared by trained and transfer tasks [32, 33], our research shows that the overlap between tasks is not enough. Particular differences between tasks may affect the degree of transfer achieved. Our findings show that our WMU training programs produced only specific improvements in WMU tasks very similar to those trained. These specific gains are compatible with the notion that a specific strategy or set of strategies had been acquired during the training. Future studies could further examine nearest transfer by attempting to identify which conditions facilitate it.

## Supporting information

S1 Appendix Questionnaire. (PDF)

S1 File Data training. (XLSX)

## References

[1] Belacchi C, Carretti B, Cornoldi C. The role of working memory and updating in Coloured Raven Matrices performance in typically developing children. European Journal of Cognitive Psychology. 2010; 22(7): 1010–1020. 10.1080/09541440903184617

[2] Chen T, Li D. The roles of working memory updating and processing speed in mediating age-related differences in fluid intelligence. Aging, Neuropsychology, and Cognition. 2007; 14(6), 631–646. 10.1080/13825580600987660 18038360

[3] Friedman NP, Miyake A, Corley RP, Young SE, DeFries JC, Hewitt JK. Not all executive functions are related to intelligence. Psychological Science. 2006; 17(2): 172–179. 10.1111/j.1467-9280.2006.01681.x 16466426

[4] Carretti B, Cornoldi C, De Beni R, Romanò M. Updating in working memory: A comparison of good and poor comprehenders. Journal of Experimental Child Psychology. 2005; 91(1): 45–66. 10.1016/j.jecp.2005.01.005 15814095

[5] Palladino P, Cornoldi C, De Beni R, Pazzaglia F. Working memory and updating processes in reading comprehension. Memory & Cognition. 2001; 29(2): 344–354. 10.3758/BF03194929 11352218

[6] Pelegrina S, Capodieci A, Carretti B, Cornoldi C. Magnitude representation and working memory updating in children with arithmetic and reading comprehension disabilities. Journal of Learning Disabilities. 2015; 48(6): 658–668. 10.1177/0022219414527480 24687221

[7] Noack H, Lövdén M, Schmiedek F. On the validity and generality of transfer effects in cognitive training research. Psychological Research. 2014; 78(6): 773–789. 10.1007/s00426-014-0564-6 24691586

[8] Jaeggi S, Buschkuehl M, Jonides J, Perrig W. Improving fluid intelligence with training on working memory. Proceedings of the National Academy of Sciences. 2008; 105(19): 6829–6833. 10.1073/pnas.0801268105 18443283 PMC2383929

[9] Li SC, Schmiedek F, Huxhold O, Röcke C, Smith J, Lindenberger U. Working memory plasticity in old age: practice gain, transfer, and maintenance. Psychology and Aging. 2008; 23(4): 731–742. 10.1037/a0014343 19140644

[10] Minear M, Brasher F, Guerrero CB, Brasher M, Moore A, Sukeena J. A simultaneous examination of two forms of working memory training: Evidence for near transfer only. Memory & Cognition. 2016; 44(7): 1014–1037. 10.3758/s13421-016-0616-9 27129921

[11] Lilienthal L, Tamez E, Shelton JT, Myerson J, Hale S. Dual *n*-back training increases the capacity of the focus of attention. Psychonomic Bulletin & Review. 2013; 20(1): 135–141. 10.3758/s13423-012-0335-6 23184506

[12] Clouter A. The effects of dual *n*-back training on the components of working memory and fluid intelligence: An individual difference approach Submitted in partial fulfilment for the requirements for the degree of masters of science. Dalhousie Universiy: Nova Scotia; 2013.

[13] Heinzel S, Schulte S, Onken J, Duong QL, Riemer TG, Heinz A, et al Working memory training improvements and gains in non-trained cognitive tasks in young and older adults. Aging, Neuropsychology, and Cognition. 2014; 21(2): 146–173. 10.1080/13825585.2013.79033 23639070

[14] Salminen T, Strobach T, Schubert T. On the impacts of working memory training on executive functioning. Frontiers in Human Neuroscience. 2012; 6: 166 10.3389/fnhum.2012.00166 22685428 PMC3368385

[15] Waris O, Soveri A, Laine M. Transfer after working memory updating training. PLoS ONE. 2015;10(9), e0138734 10.1371/journal.pone.0138734 26406319 PMC4583509

[16] Küper K, Karbach J. Increased training complexity reduces the effectiveness of brief working memory training: evidence from short-term single and dual *n*-back training interventions. Journal of Cognitive Psychology. 2016; 28(2): 199–208. 10.1080/20445911.2015.1118106

[17] Redick TS, Shipstead Z, Harrison TL, Hicks KL, Fried DE, Hambrick DZ, et al No evidence of intelligence improvement after working memory training: a randomized, placebo-controlled study. Journal of Experimental Psychology: General. 2013; 142(2): 359–379. 10.1037/a0029082 22708717

[18] Au J, Sheehan E, Tsai N, Duncan GJ, Buschkuehl M, Jaeggi SM. Improving fluid intelligence with training on working memory: a meta-analysis. Psychonomic Bulletin & Review. 2015; 22(2): 366–377. 10.3758/s13423-014-0699-x 25102926

[19] Karbach J, Verhaeghen P. Making working memory work: A meta-analysis of executive control and working memory training in older adults. Psychological Science. 2014; 25(11): 2027–2037. 10.1177/0956797614548725 25298292 PMC4381540

[20] Dougherty MR, Hamovitz T, Tidwell JW. Reevaluating the effectiveness of *n*-back training on transfer through the Bayesian lens: Support for the null. Psychonomic Bulletin & Review. 2016; 23(1): 306–316. 10.3758/s13423-015-0865-9 26082280

[21] Melby-Lervåg M, Hulme C. Is working memory training effective? A meta-analytic review. Developmental Psychology. 2013; 49(2): 270 10.1037/a0028228 22612437

[22] Melby-Lervåg M, Redick TS, Hulme C. Working memory training does not improve performance on measures of intelligence or other measures of “far transfer” evidence from a meta-analytic review. Perspectives on Psychological Science. 2016; 11(4): 512–534. 10.1177/1745691616635612 27474138 PMC4968033

[23] Simons DJ, Boot WR, Charness N, Gathercole SE, Chabris CF, Hambrick DZ, et al Do “brain-training” programs work? Psychological Science in the Public Interest. 2016; 17(3): 103–186. 10.1177/1529100616661983 27697851

[24] Soveri A, Antfolk J, Karlsson L, Salo B, Laine M. Working memory training revisited: A multi-level meta-analysis of *n*-back training studies. Psychonomic Bulletin & Review. 2017; 1–20. 10.3758/s13423-016-1217-0 28116702

[25] Bäckman L, Nyberg L, Soveri A, Johansson J, Andersson M, Dahlin E, et al Effects of working-memory training on striatal dopamine release. Science. 2011; 333(6043): 718–718. 10.1126/science.1204978 21817043

[26] Dahlin E, Neely AS, Larsson A, Bäckman L, Nyberg L. Transfer of learning after updating training mediated by the striatum. Science. 2008; 320(5882): 1510–1512. 10.1126/science.1155466 18556560

[27] Dahlin E, Nyberg L, Bäckman L, Neely AS. Plasticity of executive functioning in young and older adults: immediate training gains, transfer, and long-term maintenance. Psychology and Aging. 2008; 23(4): 720–730. 10.1037/a0014296 19140643

[28] Xiu L, Zhou R, Jiang Y. Working memory training improves emotion regulation ability: Evidence from HRV. Physiology & Behavior. 2016; 155: 25–29. 10.1016/j.physbeh.2015.12.004 26679738

[29] Linares R, Borella E, Lechuga MT, Carretti B, Pelegrina S. Training working memory updating in young adults. Psychological Research. 2017; 1–14. 10.1007/s00426-015-0726-1 28280931

[30] von Bastian C, Oberauer K. Effects and mechanisms of working memory training: a review. Psychological Research. 2014; 78: 803–820. 10.1007/s00426-013-0524-6 24213250

[31] Morrison AB, Chein JM. Does working memory training work? The promise and challenges of enhancing cognition by training working memory. Psychonomic bulletin & review. 2011; 18(1), 46–60. 10.3758/s13423-010-0034-0 21327348

[32] Jaeggi SM, Studer-Luethi B, Buschkuehl M, Su YF, Jonides J, Perrig WJ. The relationship between *n*-back performance and matrix reasoning—implications for training and transfer. Intelligence. 2010; 38(6): 625–635. 10.1016/j.intell.2010.09.001

[33] Klingberg T. Training and plasticity of working memory. Trends in Cognitive Sciences. 2010; 14(7): 317–324. 10.1016/j.tics.2010.05.002 20630350

[34] Kane MJ, Conway AR, Miura TK, Colflesh GJ. Working memory, attention control, and the *N*-back task: a question of construct validity. Journal of Experimental Psychology: Learning, Memory, and Cognition. 2007; 33(3): 615–622. 10.1037/0278-7393.33.3.615 17470009

[35] Beatty EL, Jobidon ME, Bouak F, Nakashima A, Smith I, Lam Q, et al Transfer of training from one working memory task to another: behavioural and neural evidence. Frontiers in Systems Neuroscience. 2015; 9:86 10.3389/fnsys.2015.00086 26082694 PMC4451342

[36] Jonides J. How does practice makes perfect?. Nature Neuroscience. 2004; 7(1): 10–11. 10.1038/nn0104-10 14699412

[37] Salminen T, Kühn S, Frensch PA, Schubert T. Transfer after Dual *n*-Back Training Depends on Striatal Activation Change. Journal of Neuroscience. 2016; 36(39): 10198–10213. 10.1523/JNEUROSCI.2305-15.2016 27683914 PMC6705577

[38] De Simoni C, von Bastian CC. Working memory updating and binding training: Bayesian evidence supporting the absence of transfer. Journal of Experimental Psychology General. 2018; 147(6), 829–858. 10.1037/xge0000453 29888939

[39] Shipstead Z, Redick TS, Engle RW. Is working memory training effective? Psychological Bulletin. 2012; 138(4): 628–654. 10.1037/a0027473 22409508

[40] Laine M, Fellman D, Waris O, Nyman TJ. The early effects of external and internal strategies on working memory updating training. Scientific reports. 2018; 8(1), 4045 10.1038/s41598-018-22396-5 29511316 PMC5840432

[41] Anguera JA, Bernard JA, Jaeggi SM, Buschkuehl M, Benson BL, Jennett S, et al The effects of working memory resource depletion and training on sensorimotor adaptation. Behavioural Brain Research, 2012; 228(1): 107–115. 10.1016/j.bbr.2011.11.040 22155489 PMC3264800

[42] Jaeggi SM, Buschkuehl M, Shah P, Jonides J. The role of individual differences in cognitive training and transfer. Memory & Cognition. 2014; 42(3): 464–480. 10.3758/s13421-013-0364-z 24081919

[43] Alloway TP, Bibile V, Lau G. Computerized working memory training: Can it lead to gains in cognitive skills in students? Computers in Human Behavior. 2013; 29(3): 632–638. 10.1016/j.chb.2012.10.023

[44] Penner IK, Vogt A, Stöcklin M, Gschwind L, Opwis K, Calabrese P. Computerised working memory training in healthy adults: A comparison of two different training schedules. Neuropsychological Rehabilitation. 2012; 22(5): 716–733. 10.1080/09602011.2012.686883 22671966

[45] Dunlosky J, Rawson KA, Marsh EJ, Nathan MJ, Willingham DT. Improving students’ learning with effective learning techniques: Promising directions from cognitive and educational psychology. Psychological Science in the Public Interest. 2013; 14(1), 4–58. 10.1177/1529100612453266 26173288

[46] Cepeda NJ, Coburn N, Rohrer D, Wixted JT, Mozer MC, Pashler H. Optimizing distributed practice: Theoretical analysis and practical implications. ExperimentalPsychology. 2009; 56(4), 236–246. 10.1027/1618-3169.56.4.236 19439395

[47] Schmiedek F, Hildebrandt A, Lövdén M, Wilhelm O, Lindenberger U. Complex span versus updating tasks of working memory: the gap is not that deep. Journal of Experimental Psychology: Learning, Memory, and Cognition. 2009; 35(4): 1089–1096. 10.1037/a0015730 19586272

[48] Wilhelm O, Hildebrandt A, Oberauer K. What is working memory capacity, and how can we measure it?. Frontiers in Psychology. 2013; 4:433 10.3389/fpsyg.2013.00433 23898309 PMC3721021

[49] Ecker UK. Lewandowsky S, Oberauer K, Chee AE. The components of working memory updating: an experimental decomposition and individual differences. Journal of Experimental Psychology: Learning, Memory, and Cognition. 2010; 36(1): 170–189. 10.1037/a0017891 20053053

[50] Oberauer K, Wendland M, Kliegl R. Age differences in working memory—the roles of storage and selective access. Memory & Cognition. 2003; 31(4): 563–569. 10.1037/0096-1523.30.4.689 12872872

[51] Salthouse TA, Babcock RL, Shaw RJ. Effects of adult age on structural and operational capacities in working memory. Psychology and Aging. 1991; 6(1): 118–127. 10.1037/0882-7974.6.1.118 2029360

[52] Carretti B, Cornoldi C, Pelegrina S. Which factors influence number updating in working memory? The effects of size distance and suppression. British Journal of Psychology. 2007; 98(1), 45–60. 10.1348/000712606X104175 17319050

[53] Lendínez C, Pelegrina S, Lechuga T. The distance effect in numerical memory-updating tasks. Memory & Cognition. 2011; 39(4): 675–685. 10.3758/s13421-010-0047-y 21264591

[54] Oei AC, Patterson MD. Enhancing cognition with video games: a multiple game training study. PLoS ONE. 2013; 8(3): e58546 10.1371/journal.pone.0058546 23516504 PMC3596277

[55] Russoniello CV, O’Brien K, Parks JM. The effectiveness of casual video games in improving mood and decreasing stress. Journal of Cyber Therapy and Rehabilitation. 2009; 2(1): 53–66.

[56] Stroud MJ, Whitbourne SK. Casual video games as training tools for attentional processes in everyday life. Cyberpsychology, Behavior, and Social Networking. 2015; 18(11): 654–660. 10.1089/cyber.2015.0316 26448498

[57] Whitbourne SK, Ellenberg S, Akimoto K. Reasons for playing casual video games and perceived benefits among adults 18 to 80 years old. Cyberpsychology, Behavior, and Social Networking. 2013; 16(12): 892–897. 10.1089/cyber.2012.0705 23971430

[58] Katz J, Jones MR, Shah P, Buschkuehl M, Jaeggi SM. Individual differences and motivational effects In Strobach T, Karbach J, editors. Cognitive Training: an overview of features and application. Switzerland: Springer Verlag, 2016; p. 157–166.

[59] Maraver MJ, Bajo MT, Gómez-Ariza CJ. Training on working memory and inhibitory control in young adults. Frontiers in Human Neuroscience. 2016; 10: 588 10.3389/fnhum.2016.00588 27917117 PMC5114277

[60] Carretti B, Borella E, Zavagnin M, De Beni R. Impact of metacognition and motivation on the efficacy of strategic memory training in older adults: Analysis of specific, transfer and maintenance effects. Archives of gerontology and geriatrics. 2011; 52(3), e192–e197. 10.1016/j.archger.2010.11.004 21126778

[61] Zhao X, Xu Y, Fu J, Maes JH. Are training and transfer effects of working memory updating training modulated by achievement motivation?. Memory & cognition. 2017; 46(3), 398–409. 10.3758/s13421-017-0773-5 29185201 PMC5880846

[62] Fellman D, Soveri A, Waris O, Laine M. Training of Verbal Working Memory at sentence level Fails to show Transfer. Frontiers in Communication. 2017; 2, 14 10.3389/fcomm.2017.00014

[63] Ackerman PL, Beier ME, Boyle MO. Working Memory and Intelligence: The Same or Different Constructs? Psychological Bulletin. 2005; 131(1): 30–60. 10.1037/0033-2909.131.1.30 15631550

[64] Arthur JRW, Day EA, Villado AJ, Boatman PR, Kowollik V, Bennett JRW, et al The effect of distributed practice on immediate posttraining, and long-term performance on a complex command-and-control simulation task. Human Performance. 2010; 23(5), 428–445. 10.1080/08959285.2010.515277

[65] Cattell RB, Cattell HEP. Measuring intelligence with the Culture Fair Tests. Champaign, IL: Institute for Personality and Ability Testing; 1963.

[66] Kirchner WK. Age differences in short-term retention of rapidly changing information. Journal of Experimental Psychology. 1958; 55(4): 352–358. 10.1037/h0043688 13539317

[67] Ciesielski KT, Lesnik PG, Savoy RL, Grant EP, Ahlfors SP. Developmental neural networks in children performing a Categorical *N*-Back Task. Neuroimage. 2006; 33(3): 980–990. 10.1016/j.neuroimage.2006.07.028 16997580

[68] Turner ML, Engle RW. Is working memory capacity task-dependent? Journal of Memory and Language. 1989; 28(2): 127–154. 10.1016/0749-596X(89)90040-5

[69] Cohen J. Statistical Power Analysis for the Behavioral Sciences. Lawrence Erlbaum: Hillsdale, NJ, US; 1988.

[70] Cumming G. Understanding the new statistics: Effect sizes, confidence intervals, and meta-analysis. New York: Routledge; 2012.

[71] Harrison TL, Shipstead Z, Hicks KL, Hambrick DZ, Redick TS, Engle RW. Working memory training may increase working memory capacity but not fluid intelligence. 2013; 24(12), 2409–2419. 10.1177/0956797613492984 24091548

[72] Lövdén M, Bäckman L, Lindenberger U, Schaefer S, Schmiedek F. A theoretical framework for the study of adult cognitive plasticity. Psychological Bulletin. 2010; 136(4): 659–676. 10.1037/a0020080 20565172

[73] Kimball DR, Holyoak KJ. Transfer and expertise In Tulving E, Craig FIM, editors.The Oxford handbook of memory. New York: Oxford University Press, 2000; p. 109–122.

[74] Siegler RS, Stern E. Conscious and unconscious strategy discoveries: A microgenetic analysis. Journal of Experimental Psychology: General. 1998; 127(4): 377–397. 0096-3445/98/$3.00 9857493 10.1037//0096-3445.127.4.377

[75] Miller PH, Seier WL. Strategy utilization deficiencies in children: when, where and why. Advance in child development and behaviour. 1994; 25: 107–156. 10.1016/S0065-2407(08)60051-8 7847168

[76] Baddeley AD, Longman DJA. The influence of length and frequency of training session on the rate of learning to type. Ergonomics. 1978, 21: 627–635. 10.1080/00140137808931764

[77] Foster JL, Harrison TL, Hicks KL, Draheim C, Redick TS, Engle RW. Do the effects of working memory training depend on baseline ability level? Journal of Experimental Psychology: Learning, Memory, and Cognition. 2017, 43(11): 1677–1689. 10.1037/xlm0000426 28557500

