# Supplementary material for: Nearest transfer effects of working memory training: A comparison of two programs focused on working memory updating
Source: PLoS One. 2019 Feb 13;14(2):e0211321. doi: 10.1371/journal.pone.0211321 (PMC6373913; doi:10.1371/journal.pone.0211321)
Supplement: S1 Appendix — (PDF) [file pone.0211321.s001.pdf]

- To what extent do you think the training enabled you to improve in the arithmetical updating task (which involved performing arithmetical operations on numbers in boxes and recalling the last result)? Please rate on a scale of 1 to 7.
- To what extent do you think the training enabled you to improve in the numerical *n*-back task (which involved checking whether a number presented was the same as the one seen *n* positions earlier)? Please rate on a scale of 1 to 7.
- To what extent do you think the training enabled you to improve in the categorical updating task (when you had to remember the last animal presented in each box)? Please rate on a scale of 1 to 7.
- To what extent do you think the training enabled you to improve in the categorical *n*-back task (which involved checking whether there was an animal noun *n* positions back every time the word "cat" appeared)? Please rate on a scale of 1 to 7.
- To what extent do you think the training enabled you to improve in the operation span task (when you had to check whether arithmetical operations were correct and recall a series of numbers)? Please rate on a scale of 1 to 7.
- To what extent do you think the training enabled you to improve in the Cattell test (which involved completing different logical series)? Please rate on a scale of 1 to 7.
